# SNFIMCMDA: Similarity Network Fusion and Inductive Matrix Completion for miRNA–Disease Association Prediction

**DOI:** 10.3389/fcell.2021.617569

**Published:** 2021-02-09

**Authors:** Lei Li, Zhen Gao, Chun-Hou Zheng, Yu Wang, Yu-Tian Wang, Jian-Cheng Ni

**Affiliations:** ^1^School of Software, Qufu Normal University, Qufu, China; ^2^School of Computer Science and Technology, Anhui University, Hefei, China

**Keywords:** miRNA, disease, miRNA–disease association, similarity network fusion, inductive matrix completion

## Abstract

MicroRNAs (miRNAs) that belong to non-coding RNAs are verified to be closely associated with several complicated biological processes and human diseases. In this study, we proposed a novel model that was Similarity Network Fusion and Inductive Matrix Completion for miRNA-Disease Association Prediction (SNFIMCMDA). We applied inductive matrix completion (IMC) method to acquire possible associations between miRNAs and diseases, which also could obtain corresponding correlation scores. IMC was performed based on the verified connections of miRNA–disease, miRNA similarity, and disease similarity. In addition, miRNA similarity and disease similarity were calculated by similarity network fusion, which could masterly integrate multiple data types to obtain target data. We integrated miRNA functional similarity and Gaussian interaction profile kernel similarity by similarity network fusion to obtain miRNA similarity. Similarly, disease similarity was integrated in this way. To indicate the utility and effectiveness of SNFIMCMDA, we both applied global leave-one-out cross-validation and five-fold cross-validation to validate our model. Furthermore, case studies on three significant human diseases were also implemented to prove the effectiveness of SNFIMCMDA. The results demonstrated that SNFIMCMDA was effective for prediction of possible associations of miRNA–disease.

## Introduction

MicroRNAs (miRNAs) belong to small non-coding RNAs, which effectively control the expression of their mRNA targets through RNA cleavage or translation repression (Ambros, 2004; Bartel, 2004; Ambros, 2001). In recent years, researchers have discovered various of miRNAs in many living organisms (Bruce et al., 1993; Calin and Croce, 2006). The expression of a great quantity of target genes is controlled by miRNAs, with the result that the whole miRNA pathway is an important technique for gene expression control (Xu et al., 2004; Miska, 2005; Bartel, 2009). The dysregulation of miRNAs results in progression of various diseases and conduces to developmental defects (Meola et al., 2009). Hence, identifying miRNAs that are associated with diseases is helpful in understanding the consequences of complex diseases and genetic causes. During the past few years, traditional experiments have confirmed a large number of connections of miRNA–disease (Thomson et al., 2007; Mohammadi-Yeganeh et al., 2013). Previous experimental methods such as polymerase chain reaction can reveal the relationship between miRNA and disease, but which are time-consuming and costly. Thus, revealing the more unknown relationship between miRNAs and diseases need effective experiment methods. Researchers have made every effort to achieve effective and accurate prediction methods so that future biological experiments will reliably obtain more and more reasonable and valid relationship of miRNA–disease (Han et al., 2014).

In the past period of time, a great deal of computation-based algorithms and methods were developed to predict possible relationship between miRNAs and diseases (You et al., 2017; Chen et al., 2018a). Based on an assumption that miRNAs with similar functions are highly likely to be related to diseases that were phenotypic similar and *vice versa* (Zeng et al., 2015), Jiang et al. (2010) established a novel model that identified the feasible connections of miRNA–disease by using hypergeometric distribution. However, the model had the disadvantage that it only used local similarities between two miRNAs with a large number of shared target genes. In addition, Mørk et al. (2014) constructed an miPRD model to infer the miRNA–protein connections and disease–protein connections. Then, these connections were exploited to predict the possible relationship between miRNAs and diseases. The Jaccard similarity was first introduced by Chen et al. (2018) in the model of BLHARMDA to recognize possible miRNA–disease connections. The model of BLHARMDA also introduced the system of KNN into the bipartite local model method.

Obviously, the authenticity of global network similarity measures is superior to that of local network similarity measures (Köhler et al., 2008; Zhang et al., 2014). Considering this fact, Chen et al. (2012) constructed the novel RWRMDA model to infer unknown connections of miRNA–disease. Compared to local network similarity measures, RWRMDA discovered that global network similarity was more valid to find the potential relationship between miRNAs and diseases. Therefore, the performance of previous local network-based methods was worse than RWRMDA model. However, the RWRMDA model was unsuitable for new diseases that did not associate with miRNAs. Random walk method had been proposed by many researchers so as to effectively solve this problem. Liu et al. (2016) put forward an unused method that implemented the random walk algorithm to seek miRNAs associated with diseases. The method was to construct a heterogeneous graph by integrating various similarities of diseases and miRNAs. Then, the random walk with restart method in the heterogeneous graph is applied to seek unknown connections between miRNAs and diseases. Luo and Xiao (2017) established a heterogeneous network, which was made up of the similarity of miRNA, disease semantic similarity, and verified connections of miRNA–disease. Different from the method of Liu et al. (2016), they applied the imbalanced bi-random walk method to look for diseases that related to miRNAs. Furthermore, Chen et al. (2016b) presented the WBSMDA, which integrated the various of similarities of miRNA and disease, respectively. This model also could reliably obtain the possible relationship of miRNA–disease. Another model HGIMDA was also presented by Chen et al. (2016a). The heterogeneous graph was generated by combining the verified miRNA–disease association network and the processed similarity networks of miRNA and disease in HGIMDA. It was important that an iterative equation was used in the model for the accurate prediction of potential miRNA–disease association. The model of HGIMDA performed better than previous methods, but the problem was the choice of parameters that was still not well resolved. For the purpose of inferring feasible and reasonable relationship of miRNA–disease, an identification medium was proposed by Yu et al. (2017). The medium changed the methods of maximizing information flow in existence, which consisted of functional similarity of miRNA, semantic similarity, and phenotypic similarity of disease. The verified connections and unknown connections of miRNA–disease were all adopted into a phenome–miRNAome network in this method. The NCMCMDA (Chen et al., 2020) model integrated neighborhood constraint with matrix completion algorithm to change the recovery task into an optimization problem. This model applied the fast iterative shrinkage-thresholding algorithm to recover missing interactions between miRNAs and diseases.

Recently, considerable amount of models that based on machine learning was gradually applied to expose the potential relationship of miRNA–disease. Xu et al. (2014) introduced a new method that prioritized novel disease-related miRNAs based on the miRNA target-dysregulated network (MTDN). In this model, the SVM classifier was constructed to extract the feature of network topologic information, which could effectively identify positive associations from negative associations of miRNA–disease. However, because negative samples were hard to obtain, the sets of negative samples were usually obtained by removing the pairs of positive sample sets from all pairs of miRNA–disease. In addition, Chen and Yan (2014) constructed the novel model of RLSMDA to infer potential miRNAs that were related to diseases. The association scores of miRNA–disease were effectively calculated by the model of RLSMDA. Therefore, RLSMDA could provide prediction score to new disease. Different from MTDN, RLSMDA could avert using negative miRNAs diseases associations, which could improve experimental efficiency and get more accurate results. The RBMMMDA (Chen et al., 2015) method was developed according to the restricted Boltzmann machine. RBMMMDA used the two-layer undirected graph to obviously represent the relationship of miRNA–disease. The two-layer undirected graph contained visible layer and hidden layer. RBMMMDA could gain new connections of miRNA–disease with the corresponding scores. Furthermore, another model named RKNNMDA (Chen et al., 2017) started to apply KNN method to deal with miRNAs and diseases. The support vector machine ranking model was also implemented in this method to handle these KNNs obtained by KNN method. The last ranking result of feasible connections between miRNAs and diseases was obtained by the weighted voting in this model. The disadvantage of this model was that miRNAs might associate with more known diseases owning to the bias. The BHCMDA (Zhu et al., 2020) model utilized biased heat conduction (BHC) algorithm to predict unknown connections between miRNAs and diseases through combining miRNA similarity matrix, disease similarity matrix, and miRNA–disease association matrix. The probabilistic matrix factorization (PMF) algorithm was used in IMIPMF (Ha et al., 2020) model to infer potential miRNA–disease interactions. The PMF was widely used in recommender systems, so it could effectively make use of all information to recommend miRNAs, which are strongly associated with the disease.

Because there were several limitations existing in previous models, we constructed a new model that was Similarity Network Fusion and Inductive Matrix Completion for miRNA-Disease Association Prediction (SNFIMCMDA). We used the method of similarity network fusion (SNF) to obtain similarity of miRNA, which was gained by integrating function similarity and Gaussian interaction profile (GIP) kernel similarity of miRNA. And we also used the same way to obtain the disease similarity, which was gained by integrating semantic similarity and GIP kernel similarity of disease. After collecting data and integrating similarity for miRNA and disease, we used inductive matrix completion (IMC) method to efficiently obtain possible connections of miRNA–disease. The global leave-one-out cross-validation and five-fold cross-validation were carried out to evaluate the effectiveness of SNFIMCMDA. Furthermore, colon neoplasms, lung neoplasms, and breast neoplasms were performed as case studies. As a consequence, the 44, 43, and 43 of the top 50 miRNAs inferred by SNFIMCMDA, which were validated to associate with these human diseases according to the HMDD v3.2 (Huang et al., 2019) database and dbDEMC v2.0 (Zhen et al., 2017) database, respectively. Experimental results showed that our model was effective and reliable for predicting possible relationship of miRNA–disease.

## Materials and Methods

### Human miRNA-Disease Associations

In this article, we downloaded the verified association data of miRNA–disease from HMDD v2.0 database (Li et al., 2013). There are 5,430 experimentally verified links of miRNA–disease in the known association data. Furthermore, we defined an adjacency matrix *A* ∈ *R*^*nd*×*nm*^ to describe the verified connections of miRNA–disease. There is no doubt that *nd* is defined as the amount of diseases, and *nm* is defined as the amount of miRNAs. The element *A*(*i*,*j*) is equal to 1 if disease *d*_*i*_ is validated to be related to miRNA *m*_*j*_, and 0 otherwise.

### miRNA Functional Similarity

If functions of two miRNAs are similar, they have a high probability of being related to diseases that are similar and *vice versa* (Cui, 2010; Goh et al., 2007). Obviously, the miRNA functional similarity is obtained by this assumption. miRNA functional similarity information that we obtained was downloaded from the website of http://www.cuilab.cn/files/images/cuilab/misim.zip. In addition, we indicated the matrix *MF* to stand for the miRNA functional similarity. The value of similarity between miRNA *m*_*i*_ and miRNA *m*_*j*_ is represented by element *MF*(*m*_*i*_,*m*_*j*_).

### Disease Semantic Similarity

The Directed Acyclic Network (DAG) based on the Mesh descriptor (Lipscomb, 2000) can be utilized to describe diseases. The DAG of disease *D* includes two parts: nodes and edges. The nodes in DAG represent not only the *D* itself but also ancestor nodes of *D*. The edges in DAG are applied to connect child nodes with their parent nodes directly. Then *DAG*(*D*) = (*D*,*T*(*D*),*E*(*D*)) is utilized in our article to intuitively represent the DAG of disease *D*, where *T*(*D*) and *E*(*D*) indicated the node set and edge set, respectively. The semantic score of disease *D* is calculated according to the following equation:

(1) $$D\,V\,\left(D\right)=\sum_{d\in T\,\left(D\right)}D_{D}\,\left(d\right)$$

where the contribution score of disease *d* is obtained by the following formula:

(2) $$\left\{ \begin{array}{c} D_{D}\,\left(d\right)=1\;i\,f\,d=D\\ D_{D}\,\left(d\right)=\max\left\{\Delta^{*}\,D_{D}\,\left(d^{{}^{\prime }}\right)|d^{{}^{\prime }}\in \mathrm{children}\,\mathrm{of}\,d\right\}\,\;i\,f\,d\,\text{≠}\,D \end{array}\right.$$

here, the semantic contribution factor Δ = 0.5 in our article based on previous literature (Xuan et al., 2013).

The equation to calculate semantic similarity score between disease *d*_*i*_ and disease *d*_*j*_ is as follows:

(3) $$D\,S\,\left(d_{i},d_{j}\right)=\frac{\sum_{t\in T\,\left(d_{i}\right)\cap T\,\left(d_{j}\right)}\left(D_{d_{i}}\,\left(t\right)+D_{d_{J}}\,\left(t\right)\right)}{D\,V\,\left(d_{i}\right)+D\,V\,\left(d_{j}\right)}$$

### Gaussian Interaction Profile Kernel Similarity

If functions of two miRNAs are similar, they are likely to relate to similar or same diseases and *vice versa* (Lu et al., 2008; Sanghamitra et al., 2010). Therefore, the miRNA similarity and disease similarity can use the GIP kernel similarity to represent (Chen et al., 2016; Cheng et al., 2017). First, after observing whether there is known association between disease *d*_*i*_ and each miRNA or not, the interaction profile of disease *d*_*i*_ was represented by vector *K*(*d*_*i*_). We used vector *K*(*m*_*i*_) to represent the interaction profile of miRNA *m*_*i*_ in a similar way. Then, the equations to calculate GIP kernel similarity of diseases and miRNAs are as follows:

(4) $$G\,K\,D\,\left(d_{i},d_{j}\right)=\exp\left(-\rho_{d}\,{||K\,\left(d_{i}\right)-K\,\left(d_{j}\right)||}^{2}\right)$$

(5) $$G\,K\,M\,\left(m_{i},m_{j}\right)=\exp\left(-\rho_{m}\,{||K\,\left(m_{i}\right)-K\,\left(m_{j}\right)||}^{2}\right)$$

where the GKD and GKM represent GIP kernel similarity of disease and miRNA, respectively. The ρ_*d*_ and ρ_*m*_ are utilized to regulate the bandwidths of kernel. ρ_*d*_ is calculated by normalizing the original bandwidth $\rho_{d}^{{}^{\prime }}$. The specific formula is described as follows:

(6) $$\rho_{d}=\rho_{d}^{{}^{\prime }}\,/\,\left(\frac{1}{n\,d}\,\sum_{i=1}^{n\,d}{||K\,\left(d_{i}\right)||}^{2}\right)$$

The ρ_*m*_ can be obtained in a similar way:

(7) $$\rho_{m}=\rho_{m}^{{}^{\prime }}\,/\,\left(\frac{1}{n\,m}\,\sum_{i=1}^{n\,m}{||K\,\left(m_{i}\right)||}^{2}\right)$$

### Similarity Network Fusion to Integrate Similarity

The similarity between miRNAs is calculated by functional similarity and GIP kernel similarity of miRNA, respectively. Similarly, the similarity between diseases is calculated by semantic similarity and GIP kernel similarity of disease, respectively. In this section, we introduced SNF (Wang et al., 2014) method to obtain ultimate similarity networks of disease and miRNA. The SNF method integrated similarity for disease included the following main steps. First, normalized weight matrices of disease similarity networks can be obtained by the below formulas:

(8) $$D\,S\,P\,\left(d_{i},d_{j}\right)=\left\{ \begin{array}{c} \frac{D\,S\,\left(d_{i},d_{j}\right)}{2\,\sum_{k\,\text{≠}\,i}D\,S\,\left(d_{i},d_{k}\right)}\;j\,\text{≠}\,i\\ \frac{1}{2}\;j=i \end{array}\right.$$

(9) $$K\,D\,P\,\left(d_{i},d_{j}\right)=\left\{ \begin{array}{c} \frac{G\,K\,D\,\left(d_{i},d_{j}\right)}{2\,\sum_{k\,\text{≠}\,i}G\,K\,D\,\left(d_{i},d_{k}\right)}\;j\,\text{≠}\,i\\ \frac{1}{2}\;j=i \end{array}\right.$$

where *DSP* denotes the normalized weight matrix of disease semantic similarity network, and *KDP* denotes the normalized weight matrix of GIP kernel similarity for diseases. Then, we used KNN method to calculate disease local relationship by the following two formulas:

(10) $$D\,S\,K\,\left(d_{i},d_{j}\right)=\left\{ \begin{array}{c} \frac{D\,S\,\left(d_{i},d_{j}\right)}{\sum_{k\in N_{i}}D\,S\,\left(d_{i},d_{k}\right)}\;j\in N_{i}\\ 0\;\mathrm{otherwise} \end{array}\right.$$

(11) $$K\,D\,K\,\left(d_{i},d_{j}\right)=\left\{ \begin{array}{c} \frac{G\,K\,D\,\left(d_{i},d_{j}\right)}{\sum_{k\in N_{i}}G\,K\,D\,\left(d_{i},d_{k}\right)}\;j\in N_{i}\\ 0\;\mathrm{otherwise} \end{array}\right.$$

where *N*_*i*_ denotes the number of neighbors of disease *d*_*i*_. *DSK* denotes the local relationship matrix of disease semantic similarity; *KDK* represents the local relationship matrix of GIP kernel similarity for diseases. Based on the previous literature (Wang et al., 2014), the essence of SNF method could be described as an iterative update of similar matrices. In our article, after we brought disease data into the network fusion formula of SNF, the specific process of network fusion corresponded to each data type is presented by the following equations:

(12) $$D\,S\,P\,\left(d_{i},d_{j}\right)=D\,S\,K\,{\left(d_{i},d_{j}\right)}^{*}\,K\,D\,P\,{\left(d_{i},d_{j}\right)}^{*}\,{\left(D\,S\,K\,\left(d_{i},d_{j}\right)\right)}^{T}$$

(13) $$K\,D\,P\,\left(d_{i},d_{j}\right)=K\,D\,K\,{\left(d_{i},d_{j}\right)}^{*}\,D\,S\,P\,{\left(d_{i},d_{j}\right)}^{*}\,{\left(K\,D\,K\,\left(d_{i},d_{j}\right)\right)}^{T}$$

The final similarity matrix of disease that integrated all data types is presented by the below formula:

(14) $$S_{d}\,\left(d_{i},d_{j}\right)=\frac{1}{2}\,\left(D\,S\,P\,\left(d_{i},d_{j}\right)+K\,D\,P\,\left(d_{i},d_{j}\right)\right)$$

where *S*_*d*_ denotes the finial similarity matrix of disease.

Similarity network fusion for miRNA is defined in a similar way by the following formulas:

(15) $$M\,F\,P\,\left(m_{i},m_{j}\right)=\left\{ \begin{array}{c} \frac{M\,F\,\left(m_{i},m_{j}\right)}{2\,\sum_{k\,\text{≠}\,i}M\,F\,\left(m_{i},m_{k}\right)}\;j\,\text{≠}\,i\\ \frac{1}{2}\;j=i \end{array}\right.$$

(16) $$K\,M\,P\,\left(m_{i},m_{j}\right)=\left\{ \begin{array}{c} \frac{G\,K\,M\,\left(m_{i},m_{j}\right)}{2\,\sum_{k\,\text{≠}\,i}G\,K\,M\,\left(m_{i},m_{k}\right)}\;j\,\text{≠}\,i\\ \frac{1}{2}\;j=i \end{array}\right.$$

(17) $$M\,F\,K\,\left(m_{i},m_{j}\right)=\left\{ \begin{array}{c} \frac{M\,F\,\left(m_{i},m_{j}\right)}{\sum_{k\in N_{i}}M\,F\,\left(m_{i},m_{k}\right)}\;j\in N_{i}\\ 0\;\mathrm{otherwise} \end{array}\right.$$

(18) $$K\,M\,K\,\left(m_{i},m_{j}\right)=\left\{ \begin{array}{c} \frac{G\,K\,M\,\left(m_{i},m_{j}\right)}{\sum_{k\in N_{i}}G\,K\,M\,\left(m_{i},m_{k}\right)}\;j\in N_{i}\\ 0\;\mathrm{otherwise} \end{array}\right.$$

(19) $$M\,F\,P\,\left(m_{i},m_{j}\right)=M\,F\,K\,{\left(m_{i},m_{j}\right)}^{*}\,K\,M\,P\,{\left(m_{i},m_{j}\right)}^{*}\,{\left(M\,F\,K\,\left(m_{i},m_{j}\right)\right)}^{T}$$

(20) $$K\,M\,P\,\left(m_{i},m_{j}\right)=K\,M\,K\,{\left(m_{i},m_{j}\right)}^{*}\,M\,F\,P\,{\left(m_{i},m_{j}\right)}^{*}\,{\left(M\,K\,M\,\left(m_{i},m_{j}\right)\right)}^{T}$$

(21) $$S_{m}\,\left(m_{i},m_{j}\right)=\frac{1}{2}\,\left(M\,F\,P\,\left(m_{i},m_{j}\right)+K\,M\,P\,\left(m_{i},m_{j}\right)\right)$$

where *S*_*m*_ denotes the miRNA similarity matrix.

### Inductive Matrix Completion

**FIGURE 1:**
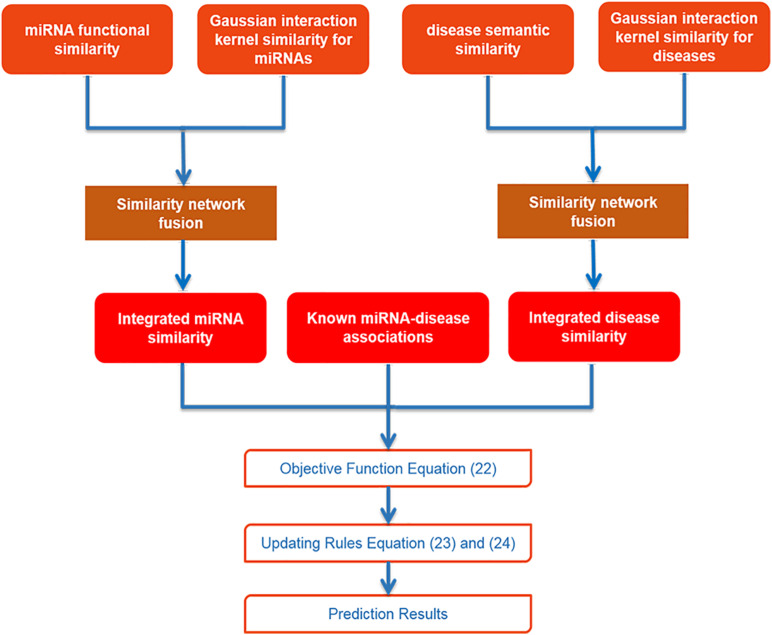
Flowchart of SNFIMCMDA model.

After collecting data and using SNF to integrate similarities for miRNA and disease, we utilized IMC method to obtain final prediction result. The specific flowchart of SNFIMCMDA is presented in Figure 1. The IMC method was employed according to the verified connection matrix of miRNA–disease *A* ∈ *R*^*nd*×*nm*^, miRNA similarity matrix *S*_*m*_ ∈ *R*^*nm*×*nm*^, and disease similarity matrix *S*_*d*_ ∈ *R*^*nd*×*nd*^. Here, the feature matrix of *nm* miRNAs was used *S*_*m*_ ∈ *R*^*nm*×*nm*^ to represent, and the feature matrix of *nd* diseases was used *S*_*d*_ ∈ *R*^*nd*×*nd*^ to represent. The feature vector of miRNA *m*_*j*_ was denoted by *S*_*m*_(*j*), and the feature vector of disease *d*_*i*_ was denoted by *S*_*d*_(*i*). Then we made *A* = *UV^T^*, where *U* ∈ *R*^*nd*×*r*^ and *V* ∈ *R*^*nm*×*r*^. Here, the *r* is desired rank that also is the same as *min*⁡(rank(*U*),*rank*(*V*)). The convergence speed of the IMC algorithm is also affected by *r*. The matrices *U* and *V* can be treated as the answers of the optimization problem as follows:

(22) ϕU,Vmin=12⁢||A-Sd⁢U⁢VT⁢SmT||F2+λ12⁢||U||F2+λ22⁢||V||F2

            s⁢u⁢c⁢h⁢t⁢h⁢a⁢t,U≥0,V≥0

where $||\cdot |{|}_{F}^{2}$ is Frobenius norm of matrix that is set to solve overfitting problems. λ_1_ and λ_2_ are equal to 1||⋅||_*F*_ that are regularization parameters.

In addition,*U* ∈ *R*^*nd*×*r*^ and *V* ∈ *R*^*nm*×*r*^ were two random dense matrices by the iterative equation to update. In our experiment, when the convergence criterion met 10^−6^, *U* and V would be obtained by iterative process. The process of IMC algorithm to obtain U and *V* are presented by the following formulas:

(23) $$V_{j\,k}\leftarrow V_{j\,k}\,\frac{{\left(S_{m}^{T}\,A^{T}\,S_{d}\,U\right)}_{j\,k}}{{\left(S_{m}^{T}\,S_{m}\,V\,U^{T}\,S_{d}^{T}\,S_{d}\,U+\lambda_{2}\,V\right)}_{j\,K}}$$

(24) $$U_{i\,k}\leftarrow U_{i\,k}\,\frac{{\left(S_{d}^{T}\,A\,S_{m}\,V\right)}_{i\,k}}{{\left(S_{d}^{T}\,S_{d}\,U\,V^{T}\,S_{m}^{T}\,S_{m}\,V+\lambda_{1}\,U\right)}_{i\,K}}$$

The *S*(*d_i_*,*m_j_*) indicates the predicted association chance between *d_i_* and *m_j_*. *S*(*d_i_*,*m_j_*) can be obtained by applying *U* and V to calculate:

(25) $$S\,\left(d_{i},m_{j}\right)=S_{d}\,\left(i\right)\,U\,V^{T}\,S_{m}\,\left(j\right)$$

Furthermore, if the feature vector of disease *newd*_*i*_ is acquired, *S*(*newd*_*i*_,*j*) can be utilized to obtain association score between this disease and any miRNA. We will realize disease *newd*_*i*_ associated with which miRNAs effectively.

## Results

### Performance Evaluation

For the purpose of affirming the accuracy of predicted result of SNFIMCMDA, we compared our model with three previous computational models: IMCMDA (Chen et al., 2018b), *GRL*_2,1_-NMF (Gao et al., 2020), and MSCHLMDA (Wu et al., 2020). Based on the verified connections of miRNA–disease that were downloaded from HMDD v2.0 database, global leave-one-out cross-validation (global LOOCV) and five-fold cross-validation (5-CV) were utilized to validate the actual performance of these computational models.

**FIGURE 2:**
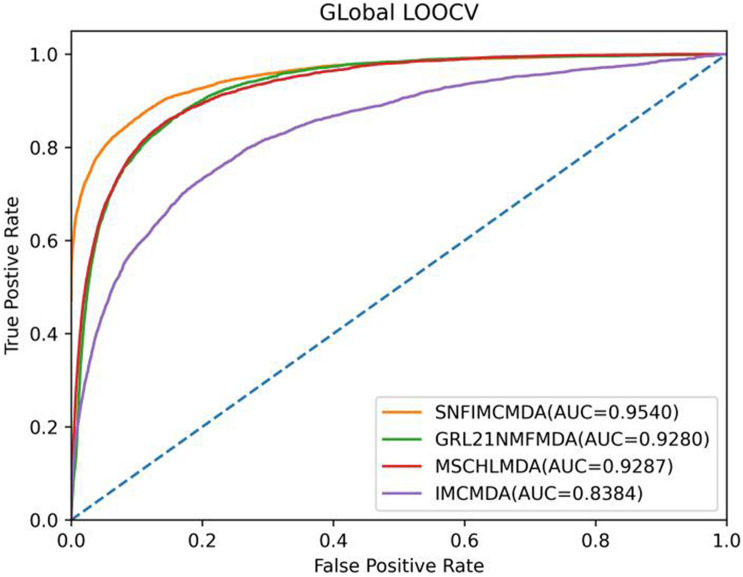
AUC of global LOOCV compared with those of IMCMDA, *GRL*_2,1_-NMF, and MSCHLMDA.

In the framework of global LOOCV, we applied the associations of miRNA–disease to train model. First, we selected each verified connection of miRNA–disease in turn for testing, whereas other experimentally confirmed associations were training sets. In addition to verified associations, there still were some connections between miRNAs and diseases without evidence that were treated as candidate samples. Then we calculated all association scores after implementing SNFIMCMDA, the test samples would obtain the predicting rankings by comparing with the candidate samples. If a given threshold was inferior to the ranking of each test sample, we thought SNFIMCMDA was valid. Furthermore, we could draw receiver operating characteristic (ROC) curve by plotting the true-positive rate against the false-positive rate. Finally, for the purpose of evaluating performance of SNFIMCMDA, we calculated the areas under ROC curve (AUCs) of all models. The ultimate result clearly indicated that the AUC values of SNFIMCMDA, IMCMDA, *GRL*_2,1_-NMF, and MSCHLMDA reached 0.9540, 0.8384, 0.9280, and 0.9287, respectively (Figure 2). Obviously, the AUC of SNFIMCMDA was higher than other methods.

**FIGURE 3:**
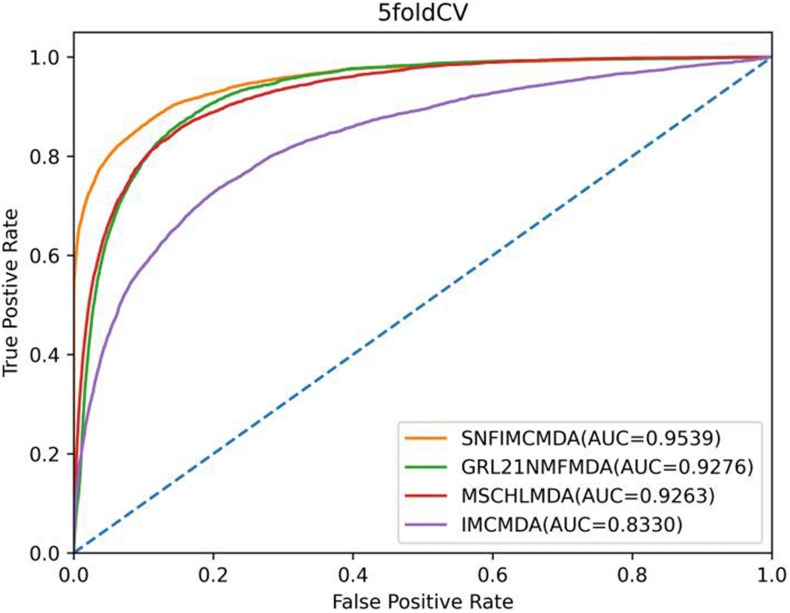
AUC of 5-CV compared with those of IMCMDA, *GRL*_2,1_-NMF, and MSCHLMDA.

In the framework of 5-CV, first, all observed connections of miRNA–disease were randomly divided into five parts; where the test set was held by each one of the five parts for each round, the training set consisted of the other four parts in turn. In addition to observed connections, there still were several connections without evidence that were treated as candidate samples. After implementing the SNFIMCMDA, we could obtain the predicted rankings of test samples compared with those of the candidate samples. Furthermore, we performed 100 times repeated segmentations on known connections, so as to avoid the possible deviations generated in the process of random sample segmentation. Finally, similar to global LOOCV, we could obtain ROC curve and AUCs of these models. The specific result indicated that the AUC values of SNFIMCMDA, IMCMDA, *GRL*_2,1_-NMF, and MSCHLMDA were 0.9539, 0.8330, 0.9276, and 0.9263, respectively (Figure 3). Obviously, the AUC of SNFIMCMDA was also higher than other methods.

### Case Study

In this article, several types of human diseases that included colon neoplasms, breast neoplasms, and lung neoplasms were applied to validate the prediction result of SNFIMCMDA. These diseases actually pose a great threat to human beings. Colon neoplasms belong to the common malignant tumor in the gastrointestinal tract (Jemal et al., 2011). There were a large amount of new cases and deaths that were caused by colon neoplasms in recent years (Thackeray et al., 2011). Several miRNAs that relate to colon neoplasms have been confirmed by a mass of biological experiments. Breast neoplasms can be regarded as a common disease in females, which has negative effects on the health of women (Koboldt et al., 2012; Liang et al., 2016). Based on clinical experiments and evidences, numerous miRNAs that are related to the breast neoplasms have been found by researchers (Fu et al., 2011; Zhu et al., 2014) in the past few years. Lung neoplasms are considered as the fastest-growing neoplasm in morbidity rate and mortality rate (Jemal et al., 2011; Carol et al., 2019). The HMDD v2.0 database was used as the training database; we applied verified associations of miRNA–disease to produce prediction results. The HMDD v3.2 database and dbDEMC v2.0 database served as validation databases utilized to validate prediction results. Furthermore, the candidate miRNAs of these diseases obtained from the SNFIMCMDA were ranked by prediction scores. Finally, we gained the top 50 miRNAs that connected to these human diseases and the 44, 43, and 43 of the top 50 miRNAs certified by HMDD v3.2 database and dbDEMC v2.0 database, respectively. The specific results are listed in Tables 1–3. In conclusion, we tested the predictive performance of SNFIMCMDA on the HMDD v2.0 database to observe whether the model had a well performance on it. As the validation results are shown in the tables, the effectiveness of SNFIMCMDA on predicting unknown interactions between miRNAs and diseases had been confirmed by the HMDD v3.2 database and dbDEMC v2.0 database.

**TABLE 1:** The top 50 potential miRNAs associated with colon neoplasms.

miRNA	Evidence	miRNA	Evidence
hsa-mir-34a	H; d	hsa-mir-133b	H; d
hsa-mir-29a	H; d	hsa-mir-206	d
hsa-mir-29b	H; d	hsa-mir-20a	H; d
hsa-mir-16	Unconfirmed	hsa-mir-30a	H; d
hsa-mir-125b	H; d	hsa-mir-31	H; d
hsa-mir-15a	H; d	hsa-mir-21	H; d
hsa-mir-221	H; d	hsa-mir-122	d
hsa-mir-181a	H; d	hsa-mir-155	H; d
hsa-mir-1	d	hsa-mir-15b	H; d
hsa-mir-223	H; d	hsa-mir-9	d
hsa-mir-29c	d	hsa-mir-7	d
hsa-mir-146a	H; d	hsa-mir-203	H; d
hsa-mir-150	H; d	hsa-mir-19b	d
hsa-mir-199a	Unconfirmed	hsa-mir-214	Unconfirmed
hsa-mir-24	H; d	hsa-mir-23a	H; d
hsa-mir-210	H; d	hsa-mir-148a	H; d
hsa-mir-181b	H; d	hsa-mir-34c	Unconfirmed
hsa-mir-222	H; d	hsa-mir-196a	H; d
hsa-mir-143	H; d	hsa-mir-132	H; d
hsa-mir-106b	H; d	hsa-mir-125a	H; d
hsa-mir-195	H; d	hsa-mir-192	H; d
hsa-mir-133a	H; d	hsa-let-7a	Unconfirmed
hsa-mir-146b	d	hsa-mir-200b	H; d
hsa-mir-142	H	hsa-mir-124	Unconfirmed
hsa-mir-92a	d	hsa-mir-18a	H; d

*H: HMDD v3.2 database; d: dbDEMC v2.0 database.*

**TABLE 2:** The top 50 potential miRNAs associated with breast neoplasms.

miRNA	Evidence	miRNA	Evidence
hsa-mir-106a	H; d	hsa-mir-363	H; d
hsa-mir-142	H	hsa-mir-196b	H; d
hsa-mir-192	H; d	hsa-mir-99b	d
hsa-mir-138	H; d	hsa-mir-98	H; d
hsa-mir-542	H	hsa-mir-552	d
hsa-mir-32	H; d	hsa-mir-186	d
hsa-mir-15b	H; d	hsa-mir-421	H; d
hsa-mir-449a	H; d	hsa-mir-144	H; d
hsa-mir-181d	d	hsa-mir-28	d
hsa-mir-150	H; d	hsa-mir-212	H; d
hsa-mir-92b	H; d	hsa-mir-130b	H; d
hsa-mir-498	d	hsa-mir-518b	Unconfirmed
hsa-mir-211	Unconfirmed	hsa-mir-494	H; d
hsa-mir-330	Unconfirmed	hsa-mir-410	H; d
hsa-mir-185	H; d	hsa-mir-512	Unconfirmed
hsa-mir-130a	H; d	hsa-mir-370	H; d
hsa-mir-518c	d	hsa-mir-181c	H; d
hsa-mir-622	d	hsa-mir-574	H; d
hsa-mir-615	Unconfirmed	hsa-mir-376a	H; d
hsa-mir-30e	H	hsa-mir-548d	H
hsa-mir-184	H; d	hsa-mir-539	d
hsa-mir-483	Unconfirmed	hsa-mir-372	H; d
hsa-mir-99a	H; d	hsa-mir-454	d
hsa-mir-502	H	hsa-mir-424	H; d
hsa-mir-630	H; d	hsa-mir-455	Unconfirmed

*H: HMDD v3.2 database; d: dbDEMC v2.0 database.*

**TABLE 3:** The top 50 potential miRNAs associated with lung neoplasms.

miRNA	Evidence	miRNA	Evidence
hsa-mir-99a	H; d	hsa-mir-424	d
hsa-mir-429	d	hsa-mir-299	H
hsa-mir-194	H; d	hsa-mir-23b	d
hsa-mir-296	Unconfirmed	hsa-mir-495	H
hsa-mir-16	H; d	hsa-mir-130b	H
hsa-mir-151a	Unconfirmed	hsa-mir-342	H; d
hsa-mir-196b	H; d	hsa-mir-141	H; d
hsa-mir-15b	d	hsa-mir-379	d
hsa-mir-10a	H; d	hsa-mir-324	H
hsa-mir-204	d	hsa-mir-663a	Unconfirmed
hsa-mir-708	d	hsa-mir-376c	d
hsa-mir-211	d	hsa-mir-520a	d
hsa-mir-122	H; d	hsa-mir-152	H; d
hsa-mir-15a	H; d	hsa-mir-608	H
hsa-mir-451a	H; d	hsa-mir-500a	Unconfirmed
hsa-mir-432	d	hsa-mir-190a	Unconfirmed
hsa-mir-217	H	hsa-mir-520d	d
hsa-mir-154	H	hsa-mir-215	H; d
hsa-mir-625	d	hsa-mir-144	H; d
hsa-mir-195	H; d	hsa-mir-184	H; d
hsa-mir-99b	d	hsa-mir-433	d
hsa-mir-28	d	hsa-mir-520c	Unconfirmed
hsa-mir-362	H	hsa-mir-367	d
hsa-mir-501	Unconfirmed	hsa-mir-520b	H; d
hsa-mir-423	H	hsa-mir-520e	H; d

*H: HMDD v3.2 database; d: dbDEMC v2.0 database.*

## Discussion

The researches for inferring possible relationship of miRNA–disease would provide deep insight into the pathogenesis of diseases and contribute to the treatment of diseases. Therefore, we constructed the novel model of SNFIMCMDA. The prediction score of each miRNA–disease pair was calculated by combining the known association between miRNAs and diseases and integrated similarities of both miRNA and disease in the SNFIMCMDA. Different from the model of IMCMDA that had been published in previous years, we made a change in integrating similarity for miRNAs and diseases. The method of SNF was used to integrate similarity in place of a previous method in IMCMDA. After adopting SNF, there was a significant improvement in the prediction results. In the framework of global LOOCV, the AUC of SNFIMCMDA was 0.9540, which was higher than 0.8330 calculated by IMCMDA. And in the framework of 5-CV, the AUC of SNFIMCMDA was 0.9539 that was also higher than 0.8330 obtained by IMCMDA. Moreover, the AUC of SNFIMCMDA performed better than other previous methods in both global LOOCV and 5-CV. Furthermore, three different human diseases were performed as case study that had effectively certified the reliable performance of the SNFIMCMDA. Therefore, SNFIMCMDA could be utilized as a reliable biological tool for extracting the most promising disease-related miRNAs, thereby enhancing our comprehension on the disease mechanisms of miRNAs and contributing to the prevention, discovery, and diagnosis of complex diseases in the future.

In our article, the model of SNFIMCMDA completed the missing association scores between miRNAs and diseases, which utilized the feature vector method to succinctly represent disease and miRNA, respectively. Furthermore, if we had the feature vector of the disease without any known associated miRNAs, the SNFIMCMDA could reliably predict this disease associated with which miRNAs and obtained the scores between them. In addition, our model belonged to semi-supervised model, so it had no use for negative samples. The obvious advantage of our model was that it only needs positive and unlabeled samples, which effectively lowered the level of difficulty of modeling to a large extent. In addition, the function of SNF was to combine different types of experimental data. We applied the SNF algorithm to combine different-type similarity data of miRNA and disease so that it makes the prediction result more reliable. Finally, the alternating gradient descent of IMC algorithm was used to find the optimal solution, which ensured the reliability of the eigenvectors of miRNA and disease.

There were some limitations that influenced the performance of SNFIMCMDA. First, the materials that we used included verified connections of miRNA–disease, miRNA function similarity, and disease semantic similarity, which may obtain noise and outliers. In addition, SNFIMCMDA used the least square error function that would cause noises and outliers. Furthermore, the model of SNFIMCMDA included several parameters. It was an obvious challenge to discover optimal parameters. Therefore, with the increasing of verified biological data, we would develop optimization strategy to improve accuracy of our model.

## Data Availability Statement

The original contributions presented in the study are included in the article/Supplementary Material, further inquiries can be directed to the corresponding author/s.

## Author Contributions

LL designed the experiment, performed the experiment, and wrote the manuscript. ZG and YW performed the experiment. C-HZ processed the data. Y-TW and J-CN revised the manuscript. All authors contributed to the article and approved the submitted version.

## Conflict of Interest

The authors declare that the research was conducted in the absence of any commercial or financial relationships that could be construed as a potential conflict of interest.

## References

[B1] Ambros V. (2001). microRNAs: tiny regulators with great potential. *Cell* 107 823–826. 10.1016/S0092-8674(01)00616-X 11779458

[B2] Ambros V. (2004). The function of animal MicroRNAs. *Nature* 431 350–355. 10.1038/nature02871 15372042

[B3] Bartel D. P. (2004). MicroRNAs: genomics, biogenesis, mechanism, and function. *Cell* 116 281–297. 10.1016/s0092-8674(04)00045-5 14744438

[B4] Bartel D. P. (2009). MicroRNAs: target recognition and regulatory functions. *Cell* 136 215–233. 10.1016/j.cell.2009.01.002 19167326 PMC3794896

[B5] Bruce W. Ilho R. Gary R. (1993). Posttranscriptional regulation of the heterochronic gene lin-14 by lin-4 mediates temporal pattern formation in *C. elegans*. *Cell* 75 855–862. 10.1016/0092-8674(93)90530-4 8252622

[B6] Calin G. A. Croce C. M. (2006). MicroRNA signatures in human cancers. *Nat. Rev. Cancer Croce* 6 857–866. 10.1038/nrc1997 17060945

[B7] Carol E. D. Kimberly D. M. Ann G. S. Ahmedin J. Rebecca L. S. (2019). Cancer statistics for African Americans, 2019. *CA A Cancer J. Clin.* 69 211–233. 10.3322/caac.21555 30762872

[B8] Chen X. Cheng J. Y. Yin J. (2018). Predicting microRNA-disease associations using bipartite local models and hubness-aware regression. *RNA Biol.* 15 1192–1205. 10.1080/15476286.2018.1517010 30196756 PMC6284580

[B9] Chen X. Gong Y. Zhang D. H. You Z. H. Li Z. W. (2018a). DRMDA: deep representations-based miRNA–disease association prediction. *J. Cell Mol. Med.* 22 472–485. 10.1111/jcmm.13336 28857494 PMC5742725

[B10] Chen X. Wang L. Qu J. Guan N. N. Li J. Q. (2018b). Predicting miRNA–disease association based on inductive matrix completion. *Bioinformatics* 24 4256–4265. 10.1093/bioinformatics/bty503 29939227

[B11] Chen X. Huang Y. A. You Z. H. Yan G. Y. Wang X. S. (2016). A novel approach based on KATZ measure to predict associations of human microbiota with non-infectious diseases. *Bioinformatics* 33 733–739. 10.1093/bioinformatics/btw715 28025197

[B12] Chen X. Liu M. X. Yan G. Y. (2012). RWRMDA: predicting novel human microRNA-disease associations. *Mol. Biosyst.* 8 2792–2798. 10.1039/c2mb25180a 22875290

[B13] Chen X. Sun L. G. Zhao Y. (2020). NCMCMDA: miRNA-disease association prediction through neighborhood constraint matrix completion. *Brief. Bioinform.* S2, 1–12. 10.1093/bib/bbz159 31927572

[B14] Chen X. Wu Q. F. Yan G. Y. (2017). RKNNMDA: ranking-based KNN for MiRNA-disease association prediction. *RNA Biol.* 14 952–962. 10.1080/15476286.2017.1312226 28421868 PMC5546566

[B15] Chen X. Yan C. C. You Z. H. Huang Y. A. Yan G. Y. (2016a). HGIMDA: heterogeneous graph inference for miRNA-disease association prediction. *Oncotarget* 7 65257–65269. 10.18632/oncotarget.11251 27533456 PMC5323153

[B16] Chen X. Yan C. C. Zhang X. You Z. H. Deng L. Liu Y. (2016b). WBSMDA: within and between score for MiRNA-disease association prediction. *Sci. Rep.* 6:21106. 10.1038/srep21106 26880032 PMC4754743

[B17] Chen X. Yan C. C. Zhang X. Li Z. Deng L. Zhang Y. (2015). RBMMMDA: predicting multiple types of disease-microRNA associations. *Entific Rep.* 5:13877. 10.1038/srep13877 26347258 PMC4561957

[B18] Chen X. Yan G. Y. (2014). Semi-supervised learning for potential human microRNA-disease associations inference. *Entific Rep.* 4:5501. 10.1038/srep05501 24975600 PMC4074792

[B19] Cheng Y. Wang J. Lan W. Wu F. X. Pan Y. (2017). SDTRLS: predicting drug-target interactions for complex diseases based on chemical substructures. *Complexity* 2017 1–10. 10.1155/2017/2713280

[B20] Cui Q. (2010). Inferring the human microRNA functional similarity and functional network based on microRNA-associated diseases. *Bioinformatics* 26 1644–1650. 10.1093/bioinformatics/btq241 20439255

[B21] Fu S. W. Chen L. Man Y. G. (2011). miRNA biomarkers in breast cancer detection and management. *J. Cancer* 2 116–122. 10.7150/jca.2.116 21479130 PMC3072617

[B22] Gao Z. Wang Y. Wu Q. Ni J. Zheng C. (2020). Graph regularized L2,1-nonnegative matrix factorization for miRNA-disease association prediction. *BMC Bioinformatics* 21:61. 10.1186/s12859-020-3409-x 32070280 PMC7029547

[B23] Goh K. I. Cusick M. E. Valle D. Childs B. Vidal M. Barabási A. L. (2007). The human disease network. *Proc. Natl. Acad. Sci. U.S.A.* 104 8685–8690. 10.1073/pnas.0701361104 17502601 PMC1885563

[B24] Ha J. Park C. H. Park C. Y. Park S. (2020). IMIPMF: inferring miRNA-disease interactions using probabilistic matrix factorization. *J. Biomed. Inform.* 102:103358. 10.1016/j.jbi.2019.103358 31857202

[B25] Han K. Xuan P. Ding J. Zhao Z. J. Zhong Y. L. (2014). Prediction of disease-related microRNAs by incorporating functional similarity and common association information. *Genet. Mol. Res. Gmr.* 13 2009–2019. 10.4238/2014.March.24.5 24737426

[B26] Huang Z. Shi J. Gao Y. Cui C. Zhang S. Li J. (2019). HMDD v3.0: a database for experimentally supported human microRNA–disease associations. *Nucleic Acids Res.* 47 D1013–D1017. 10.1093/nar/gky1010 30364956 PMC6323994

[B27] Jemal A. Bray F. Center M. M. Ferlay J. Ward E. Forman D. (2011). Global cancer statistics. *CA Cancer J. Clin.* 61 69–90. 10.3322/caac.20107 21296855

[B28] Jiang Q. Hao Y. Wang G. Juan L. Wang Y. (2010). Prioritization of disease microRNAs through a human phenome-microRNAome network. *BMC Syst. Biol.* S2:4. 10.1186/1752-0509-4-S1-S2 20522252 PMC2880408

[B29] Koboldt D. C. Fulton R. S. Schmidt H. Dooling D. J. Ding L. (2012). Comprehensive molecular portraits of human breast tumors. *Nature* 490 61–70. 10.1038/nature11412 23000897 PMC3465532

[B30] Köhler S. Bauer S. Horn D. Robinson P. N. (2008). Walking the Interactome for Prioritization of Candidate Disease Genes. *Am. J. Hum. Genet.* 82, 949–958. 10.1016/j.ajhg.2008.02.013 18371930 PMC2427257

[B31] Li Y. Qiu C. Tu J. Geng B. Yang J. Jiang T. (2013). HMDD v2.0: a database for experimentally supported human microRNA and disease associations. *Nucleic Acids Res.* 42 D1070–D1074. 10.1093/nar/gkt1023 24194601 PMC3964961

[B32] Liang C. Zhang H. Mei Q. (2016). A discriminative feature extraction approach for tumor classification using gene expression data. *Curr. Bioinform.* 11:1 10.2174/1574893611666160728114747

[B33] Lipscomb C. E. (2000). Medical subject headings (MeSH). *Bull. Med. Libr. Assoc.* 88 265–266. 10928714 PMC35238

[B34] Liu Y. Zeng X. He Z. Zou Q. (2016). Inferring MicroRNA-disease associations by random walk on a heterogeneous network with multiple data sources. *IEEE/ACM Trans. Comput. Biol. Bioinform.* 4:1. 10.1109/TCBB.2016.2550432 27076459

[B35] Lu M. Zhang Q. Deng M. Miao J. Guo Y. Gao W. (2008). An analysis of human MicroRNA and disease associations. *PLoS One* 3:e3420. 10.1371/journal.pone.0003420 18923704 PMC2559869

[B36] Luo J. Xiao Q. (2017). A novel approach for predicting microRNA-disease associations by unbalanced bi-random walk on heterogeneous network. *J. Biomed. Inform.* 66 194–203. 10.1016/j.jbi.2017.01.008 28104458

[B37] Meola N. Gennarino V. Banfi S. (2009). microRNAs and genetic diseases. *Pathogenetics* 2:7. 10.1186/1755-8417-2-7 19889204 PMC2778645

[B38] Miska E. A. (2005). How microRNAs control cell division, differentiation and death. *Curr. Opin. Genet. Dev.* 15 563–568. 10.1016/j.gde.2005.08.005 16099643

[B39] Mohammadi-Yeganeh S. Paryan M. Mirab Samiee S. Soleimani M. Arefian E. Azadmanesh K. (2013). Development of a robust, low cost stem-loop real-time quantification PCR technique for miRNA expression. *Mol. Biol. Rep.* 40 3665–3674. 10.1007/s11033-012-2442-x 23307300

[B40] Mørk S. Sune P. F. Albert P. C. Jan G. Juhl J. L. (2014). Protein-driven inference of miRNA-disease associations. *Bioinformatics* 30 392–397. 10.1093/bioinformatics/btt677 24273243 PMC3904518

[B41] Sanghamitra B. Ramkrishna M. Ujjwal M. Zhang M. Q. (2010). Development of the human cancer microRNA network. *Silence* 1:6. 10.1186/1758-907X-1-6 20226080 PMC2835996

[B42] Thackeray E. W. Charatcharoenwitthaya P. Elfaki D. Sinakos E. Lindor K. D. (2011). Colon neoplasms develop early in the course of inflammatory bowel disease and primary sclerosing cholangitis. *Clin. Gastroenterol. Hepatol.* 9 52–56. 10.1016/j.cgh.2010.09.020 20920596

[B43] Thomson J. M. Parker J. S. Hammond S. M. (2007). Microarray analysis of miRNA gene expression. *Methods Enzymol.* 427:107 10.1016/S0076-6879(07)27006-5 17720481

[B44] Wang B. Mezlini A. M. Demir F. Fiume M. Tu Z. Brudno M. (2014). Similarity network fusion for aggregating data types on a genomic scale. *Nat. Methods* 11 333–337. 10.1038/nmeth.2810 24464287

[B45] Wu Q. Wang Y. Gao Z. Ni J. Zheng C. (2020). MSCHLMDA: multi-similarity based combinative hypergraph learning for predicting MiRNA-disease association. *Front. Genet.* 11:354. 10.3389/fgene.2020.00354 32351545 PMC7174776

[B46] Xu C. Ping Y. Li X. Zhao H. Wang L. Fan H. (2014). Prioritizing candidate disease miRNAs by integrating phenotype associations of multiple diseases with matched miRNA and mRNA expression profiles. *Mol. Biosyst.* 10 2800–2809. 10.1039/c4mb00353e 25099736

[B47] Xu P. Guo M. Hay B. A. (2004). MicroRNAs and the regulation of cell death. *Trends Genet.* 20 617–624. 10.1016/j.tig.2004.09.010 15522457

[B48] Xuan P. Han K. Guo M. Guo Y. Li J. Ding J. (2013). Prediction of microRNAs associated with human diseases based on weighted k most similar neighbors. *PLoS One* 8:e70204 10.1371/annotation/28592478-72f5-4937-919b-b2342d6ceda0 PMC3738541 23950912

[B49] You Z. Huang Z. A. Zhu Z. X. Yan G. Y. Chen X. (2017). PBMDA: a novel and effective path-based computational model for miRNA-disease association prediction. *PLoS Comput. Biol.* 13:e1005455. 10.1371/journal.pcbi.1005455 28339468 PMC5384769

[B50] Yu H. Chen X. Lu L. (2017). Large-scale prediction of microRNA-disease associations by combinatorial prioritization algorithm. *Entific Rep.* 7:43792. 10.1038/srep43792 28317855 PMC5357838

[B51] Zeng X. Zhang X. Zou Q. (2015). Integrative approaches for predicting microRNA function and prioritizing disease-related microRNA using biological interaction networks. *Brief. Bioinform.* 17 193–203. 10.1093/bib/bbv033 26059461

[B52] Zhang H. Cao L. Gao S. (2014). A locality correlation preserving support vector machine. *Pattern Recogn.* 47 3168–3178. 10.1016/j.patcog.2014.04.004

[B53] Zhen Y. Wu L. Wang A. Tang W. Zhao Y. Zhao H. (2017). dbDEMC 2.0: updated database of differentially expressed miRNAs in human cancers. *Nucleic Acids Res.* 45 D812–D818. 10.1093/nar/gkw1079 27899556 PMC5210560

[B54] Zhu J. Zheng Z. Wang J. Sun J. Wang P. Cheng X. (2014). Different miRNA expression profiles between human breast cancer tumors and serum. *Front. Genet.* 5:149. 10.3389/fgene.2014.00149 24904649 PMC4033838

[B55] Zhu X. Wang X. Zhao H. Pei T. Kuang L. Wang L. (2020). BHCMDA: a new biased heat conduction based method for potential MiRNA-disease association prediction. *Front. Genet.* 11:384 10.3389/fgene.2020.00384 PMC7212362 32425979

