# Supplementary material for: SNFIMCMDA: Similarity Network Fusion and Inductive Matrix Completion for miRNA–Disease Association Prediction
Source: Front Cell Dev Biol. 2021 Feb 9;9:617569. doi: 10.3389/fcell.2021.617569 (PMC7900415; doi:10.3389/fcell.2021.617569)
Supplement: Supplementary file 1 [file Table_1.DOCX]

Supplementary Material

# Supplementary Table

We applied SNFIMCMDA to prioritize all the candidate miRNA-disease pairs based on all the known miRNA-disease associations in HMDD v2.0 database as training samples. This prediction result is released for further experimental validation and research.
